# Association Between Preoperative Sleep Disturbance and Postoperative Delirium in Adults Undergoing Elective Surgery

**DOI:** 10.7759/cureus.85114

**Published:** 2025-05-31

**Authors:** Yashar Mashayekhi, Auj Ul Huda Ali, Muhammad Noman Shafique, Tariq Akbari, Falak Naz, Muneeba Shaukat, Moeed Akbar Malik, Pasbaan Rehmat, Mehreen Junaid, Mahnoor Fahim, Tamer Mubarak

**Affiliations:** 1 Medicine, Leicester University Hospital, Leicester, GBR; 2 Medicine, Rawalpindi Medical University, Rawalpindi, PAK; 3 Medicine, King's Mill Hospital, Sutton-in-Ashfield, GBR; 4 Internal Medicine, Mindwave Research Center, Islamabad, PAK; 5 Internal Medicine, Chandka Medical College, Larkana, PAK; 6 Medicine, King Edward Medical University, Lahore, PAK; 7 Medicine, Shahina Jamil Teaching Hospital, Abbottabad, PAK; 8 Medicine, Shifa College of Medicine, Islamabad, PAK; 9 Medicine, CMH Lahore Medical College and Institute of Dentistry, Lahore, PAK; 10 Medicine, Army Medical College, Rawalpindi, PAK; 11 Internal Medicine, Rashid Hospital, Dubai, ARE

**Keywords:** elective surgery, older adults, postoperative delirium, preoperative risk factors, sleep disturbance

## Abstract

Background

Postoperative delirium (POD) is a frequent and severe neuropsychiatric complication in surgical patients, often leading to prolonged hospitalization, cognitive decline, and increased healthcare costs. Emerging evidence suggests that preoperative sleep disturbances may significantly contribute to POD risk. This study investigates the association between preoperative sleep quality and POD among adults undergoing elective surgery.

Methods

A prospective cohort study was undertaken in Islamabad, Pakistan, between December 8, 2024, and March 18, 2025, among 300 patients aged 40 years and older undergoing elective surgery. Pittsburgh Sleep Quality Index (PSQI) was administered preoperatively, whereas POD and cognitive functioning were measured by the Confusion Assessment Method (CAM) and the Mini-Mental State Examination (MMSE), respectively. Statistical analysis involved Pearson correlations, independent t-tests, ANOVA, chi-square tests, and multiple regression, which was conducted using SPSS version 26.0 (IBM SPSS Statistics for Windows, IBM Corp., Armonk, NY).

Results

Poor quality of sleep in the preoperative period correlated significantly with more delirium symptoms (r = 0.374, p < 0.001) and lower cognitive function (r = 0.589, p < 0.001). Those with existing cognitive dysfunction demonstrated poorer sleep patterns and MMSE scores. The only notable exceptions here were for both extremely short (two to four hours) and excessively long (eight to 10 hours) sleep durations, which were each accompanied by increased confusion symptoms. Regression analysis showed that PSQI and MMSE scores significantly predicted CAM results (p < 0.05). Moreover, chronic illnesses like respiratory diseases and cognitive conditions were associated with disturbed sleep and decreased perceived restfulness.

Conclusion

Preoperative sleep disturbances are strongly related to a high risk of POD and reduced cognitive function in adults with elective surgery. Systematic preoperative screening for sleep quality and the use of targeted sleep interventions could provide effective strategies for POD incidence reduction and optimization of postoperative outcomes. Longitudinal studies are advised in the future to confirm these findings and inform improvements in perioperative care.

## Introduction

Delirium can be defined as a dysfunctional state of the brain in which there is a loss of cognitive function paired with diminished consciousness, impairment of perception, and the inability to concentrate on regular tasks, along with attention deficit and inhibition of senses [1]. Postoperative delirium, commonly abbreviated as POD, is a highly critical medical condition requiring urgent intervention and proper neuropsychiatric rehabilitation. The incidence of POD continues to rise in adults undergoing elective surgery and is mainly dependent on the type of surgery the patient is undergoing. The incidence reaches about 40% in patients undergoing primary surgical operations, such as cardiac interventions [2], whereas it is around 5-10% in patients undergoing relatively low-risk operations, such as arthroplasty.

Delirium presents in three forms: hyperactive, hypoactive, and mixed. Definitions of these forms vary between studies, with some emphasizing motoric features and others arousal aspects [3]. In these three, hypoactive is the most common type and presents as a consistent presentation in 28% of cases, being even more prevalent when variable presentations are considered [4]. However, an overwhelming percentage of patients present with hypoactive delirium, which is prone to being missed in clinical examinations [5]. The development of POD is preceded by various precipitating factors and the patient’s general physical and health profile before undergoing elective surgical procedures. These precipitating factors include the use of multiple medications, also known as polypharmacy, the length of the patient’s hospital stay, prior sleep disturbances, the amount of blood lost during surgery, the level of pain, the preoperative hemoglobin level, and the patient's nutritional status [6]. Evidence also suggests that the development of urinary tract infections (UTIs) can contribute to the incidence of delirium in the aging population [7].

Delirium can occur frequently and be fatal in critical patients, which translates into increased mortality rates and prolonged lengths of stay. Pharmacologic modalities, such as haloperidol, have proved ineffective in the prevention of ICU delirium; however, evidence concerning earlier nonpharmacological models is scant. Still, the postoperative impact of preoperative sleep disturbances on delirium in elective surgery patients needs to be studied [8]. The disruption of sleep patterns, together with sleep fragmentation, contributes to POD onset in adults who undergo elective surgeries. Sleep disturbance is a group of disorders characterized by the inability to initiate and maintain the body's natural sleep cycle for extended periods [9]. Poor quality of sleep and the presence of sleep disorders have been widely associated with the development of neurocognitive decline, cardiovascular, as well as respiratory problems [10].

It has been postulated that patients who are regularly affected by sleep disorders are more likely to develop POD than their counterparts [11]. The prevalence rate of postoperative sleep disturbance in patients who have undergone major surgical procedures and were, in turn, given general anesthesia, consequently, is reported to range from 15% to an alarming 72% [12], thus highlighting the severity of this problem and the after-effects on the adult surgical population.

Using several non-pharmacologic methods has proved useful in cutting down on delirium, mainly outside of the ICU. Such techniques put a strong focus on preventing insomnia by dealing with things that can be modified, including sleep disturbances [13]. Similarly, the role of pharmacological interventions, such as the administration of cholinergic stimulants or steroids to reduce POD in postoperative patients, is understudied and has not yielded favorable results [14].

Despite its clinical relevance, limited studies have explicitly focused on the relationship between preoperative sleep quality and the risk of POD in surgical patients. Investigating this association is essential for identifying at-risk individuals and improving perioperative care strategies to reduce postoperative complications.

Rationale

POD is a frequent and severe complication in adult patients after elective surgery, with postoperative prolonged hospitalization, health costs, and long-term cognitive impairment. However, its multifactorial etiology, sleep disturbance, has been identified as a potentially modifiable risk factor. However, the specific contribution of preoperative sleep disturbance to POD prediction is under-investigated. By examining this relationship, the research seeks to determine if enhancing sleep quality before surgery could be used as a preventive measure, thus minimizing the occurrence of POD and enhancing postoperative outcomes among patients.

Objectives

This research explores how preoperative sleep disorders affect POD development in surgical patients aged 40 and above. It also aims to establish the prevalence of sleep disturbances before surgery in this population and their relationship to POD symptom severity while accounting for demographic and clinical aspects.

## Materials and methods

Study design

This was a prospective cohort study designed to evaluate the relationship between preoperative sleep problems and POD in adult patients undergoing elective surgery. Data were obtained related to how much sleep each patient had before surgery, and were assessed preoperatively and followed up 24 to 48 hours after the operation to evaluate the presence of delirium. This study plan had a direct temporal relation between exposure and outcome, which maximized the validity of evaluated associations. This study was carried out at the Ahmed Medical Complex in Islamabad, Pakistan. Informed consent was taken from all the participants, and strict precautions were followed to provide anonymity and confidentiality during the study.

Sample size and technique

The study utilized an infinite population formula to calculate the sample size since the exact population total remained unknown. The formula is given below:

Sample size = Z2 × p (1-p) / d2

Where Z is the confidence interval level statistic, p is the expected prevalence or proportion, and d is the margin of error or precision. Thus, for 95% confidence, the value of Z is 1.96, and the margin of error is 0.05. The prevalence from a similar study in Pakistan with almost similar participants was used for the expected prevalence, which was 40.7%. Thus, the value of p was 0.407. Using these values, the sample size comes out to be 371 [15].

A total of 300 participants were included in the study. While limited by time, resources, and participant availability, this sample size meets the minimum requirement for statistical analysis and is considered adequate for a cohort design. The participants were recruited from the general surgical, orthopedic, and gynecology wards of Islamabad-based tertiary care hospitals. Elective procedures were surgeries like hernia repair, cholecystectomy, joint replacement, and hysterectomy. These departments were chosen because they routinely undertake planned operations and provide proper preoperative and postoperative evaluations.

A well-defined set of selection criteria was applied to ensure relevance and consistency. These are presented in Table 1.

**Table 1:** Inclusion and Exclusion Criteria for Participants Undergoing Elective Surgery in the Study PSQI = Pittsburgh Sleep Quality Index

Inclusion criteria	Exclusion criteria
Adults aged 40 years and above undergo elective surgery	Patients with known psychiatric or neurological disorders
Preoperative patients reporting sleep disturbances	Emergency surgical patients
Ability to understand and provide informed consent	Patients taking medications known to affect cognition
Completed preoperative sleep quality assessment using PSQI	Patients with a previous diagnosis of delirium
Medically stable and scheduled for routine postoperative care	Incomplete or missing questionnaire data

Data collection and study tools

Data collection was done using a non-probability sampling technique. Data were collected from participants using a questionnaire that had four parts. Preoperatively, information about sleep quality and other related data was gathered as part of the baseline assessment to ensure that sleep disturbance occurred before surgery. Postoperative assessment was done within 24 to 48 hours after surgery, once the patients had stabilized and were conscious in the postoperative recovery unit or surgical ward.

Demographics

The first part of the questionnaire consisted of demographics and asked about age, gender, educational status, and general characteristics of patients.

Pittsburgh Sleep Quality Index (PSQI)

The second section employed the PSQI scale that Buysse et al. established in 1989. Sleep disturbances and quality assessment were conducted through the self-rated PSQI scale. Good sleep quality corresponds to PSQI scores below five, yet a score above five indicates poor sleep quality and worse sleep disturbances [16].

Confusion Assessment Method (CAM)

The third part of the questionnaire used the self-rated CAM in English, which was established by Inouye and others in 1990, to identify any symptoms of confusion and delirium. The scale has a sensitivity and specificity of 94-100% and 90-95%, respectively. The CAM consists of four parts, namely, A (onset), B (inattention), C (thinking), and D (consciousness). A diagnosis of delirium is supported by features in both A and B, along with features of either C or D. CAM, in this research, was delivered by trained research staff who had prior training in its application to achieve consistency and accuracy in measurements. Assessments were made 24-48 hours after surgery when patients were stable and alert [17].

Mini-Mental State Examination (MMSE)

The final part of the questionnaire used the self-rated screening MMSE in English, developed by Folstein and colleagues in 1975, to identify cognitive impairment. A score of 24-30 meant no cognitive impairment, while a score of 18-23 and 0-17 indicated moderate and severe cognitive impairment, respectively. It has a good degree of internal consistency (Cronbach's alpha ranging from 0.70 to 0.90) and is valid across different populations and clinical conditions. The MMSE is not a self-rated test. Administration of the MMSE must be conducted by a trained interviewer. Testing lasts for about five to 10 minutes. The tool is in the public domain and translated and validated into various languages, such as English and Urdu, for populations speaking those languages. This study administered the English version, considering the literacy level of the participant population [18].

Statistical analysis

The researchers conducted the statistical analysis using SPSS version 26.0 (IBM SPSS Statistics for Windows, IBM Corp., Armonk, NY). For demographics and general characteristics of the participants, frequencies and percentages were calculated using descriptive statistics. The Pearson correlation test established a correlation between sleep quality, confusion symptoms, and cognitive function. A t-test analysis determined the average scores on PSQI, CAM, and MMSE according to whether patients used sleep meds or experienced delirium before the study. The ANOVA test was used to compare the mean differences between PSQI, CAM, and MMSE in the context of difficulty falling asleep, feeling rested upon waking, and sleep duration. A multiple regression model using a 95% confidence interval was applied to assess poor sleep quality and cognitive dysfunction as predictors of confusion symptoms. The chi-square test was used to assess the association of feeling rested upon waking and sleep duration per night with difficulty sleeping and pre-existing medical conditions. A p-value of less than 0.05 was considered statistically significant during the study.

Ethical considerations

Ethical approval to conduct this study was provided by the Ethical Review Board of Mindwave Research Center, Islamabad, Pakistan (IRB-2024-0062). All participants were informed about the study's aims and methods before taking part. Written informed consent was taken from all participants. Participation was completely voluntary, and participants could withdraw at any time without penalty. There was confidentiality and anonymity rigorously maintained in the entire research. Identifiers for personal information were replaced by unique participant codes, and only de-identified data were utilized for the analysis of the collected data. Personal information was utilized solely for research purposes and managed in line with established ethical principles. Data collection was carried out between December 8, 2024, and March 18, 2025.

## Results

Table 2 presents the demographic and clinical profile of 300 participants aged between 40 and 70 years. Most participants (226 individuals, 75%) were aged 40-50 years, followed by 55 (18%) in the 51-60 age group and 19 (6%) aged 61-70 years. Males represented the largest gender group (181, 60%), followed by females (92, 31%), while 27 participants (9%) preferred not to disclose their gender.

**Table 2:** Demographic Characteristics of Participants (n = 300) f = frequency, % = percentage

Variable	f	%
Age		
40-50 years	226	75
51-60 years	55	18
61-70 years	19	6
Gender		
Male	181	60
Female	92	31
Prefer not to say	27	9
Marital status		
Single	120	40
Married	97	32
Divorced	63	21
Widowed	20	7
Educational level		
No formal education	84	28
Primary	80	27
Secondary	58	19
Higher secondary	55	18
Graduate	18	6
Postgraduate	5	2
Living arrangement		
Alone	103	34
With spouse	108	36
With family/relatives	68	23
In an assisted living facility	21	7
History of sleep problems		
Yes	198	66
No	102	34
Use of sleep medication		
Yes	215	72
No	85	28
Pre-existing medical conditions		
Hypertension	99	33
Diabetes	93	31
Cardiac disease	44	15
Respiratory disease	34	11
Cognitive impairment/dementia	18	6
Depression/anxiety	12	4
History of delirium or cognitive impairment		
Yes	220	73
No	80	27
Smoking status		
Current smoker	115	38
Former smoker	125	42
Never smoked	60	20
Alcohol consumption		
Yes	195	65
No	105	35
Difficulty in falling asleep		
Never	134	45
Occasionally	108	36
Frequently	41	14
Always	17	6
Waking up at night		
Never	53	18
Occasionally	145	48
Frequently	73	24
Always	29	10
Feeling rested upon waking		
Never	17	6
Rarely	26	9
Sometimes	63	21
Always	77	26
Often	117	39

In terms of marital status, 120 individuals (40%) were single, 97 (32%) were married, 63 (21%) divorced, and 20 (7%) widowed. Regarding educational level, 84 participants (28%) had no formal education, 80 (27%) had primary education, 58 (19%) had completed secondary education, 55 (18%) had higher secondary education, 18 (6%) were graduates, and only five (2%) held postgraduate degrees.

Living arrangements showed that 108 participants (36%) lived with a spouse, 103 (34%) lived alone, 68 (23%) with family or relatives, and 21 (7%) in assisted living facilities. A history of sleep problems was reported by 198 participants (66%), and 215 (72%) used sleep medications. Concerning pre-existing medical conditions, hypertension was the most common (99, 33%), followed by diabetes (93, 31%), cardiac disease (44, 15%), respiratory disease (34, 11%), cognitive impairment or dementia (18, 6%), and depression or anxiety (12, 4%). Additionally, 220 participants (73%) had a history of delirium or cognitive impairment.

The survey revealed 115 participants (38%) who were active smokers, along with 125 former smokers (42%), while 60 individuals (20%) stated they had never smoked. Alcohol consumption was reported by 195 participants (65%), while 105 (35%) denied alcohol use.

Sleep-related behavior showed that 17 participants (6%) and 41 (14%) frequently had difficulty falling asleep, while 108 (36%) reported occasional difficulty. Night awakenings were every day, with 29 participants (10%) constantly and 73 (24%) frequently waking up at night. Only 77 participants (26%) always felt rested upon waking, while 117 (39%) reported often, 63 (21%) sometimes, 26 (9%) rarely, and 17 (6%) never felt rested.

Table 3 demonstrates the interrelationships among participants' sleep quality, cognitive status, and delirium symptoms. The PSQI scores were positively associated with the CAM scores (r = 0.374, p < 0.01) at a significant level that was both moderate. This indicates that poor sleep quality leads to more delirium symptoms. A stronger, significant positive correlation was observed between PSQI and MMSE scores (r = 0.589, p < 0.01), suggesting that poorer sleep quality was also moderately associated with reduced cognitive function.

**Table 3:** Intercorrelations Between the Study Variables *p < 0.001 considered significant; correlation = Pearson correlation

Variable	Pittsburgh Sleep Quality Index	Confused Assessment Method	Mini-Mental State Examination
Pittsburgh Sleep Quality Index	-	0.374^*^	0.589^*^
Confused Assessment Method	0.374^*^	-	0.494^*^
Mini-Mental State Examination	0.589^*^	0.494^*^	-

CAM and MMSE scores also showed a positive correlation (r = 0.494, p < 0.01), reflecting that higher confusion symptoms were moderately linked to lower cognitive performance. These findings highlight significant interdependence between sleep disturbances, cognitive impairment, and delirium risk in the studied population.

Table 4 compares the scores on sleep quality, delirium symptoms, and cognitive function between participants who reported using sleep medication and those who did not. Participants who used sleep medication (M = 47.7, SD = 7.8) had significantly better sleep quality (lower PSQI scores) compared to non-users (M = 52.1, SD = 9.6), with a statistically significant difference (t = -4.109, p < 0.001, 95% CI = -6.530 to -2.301). The sleep quality difference demonstrated a moderate effect size through Cohen's d = 0.53. Similarly, CAM scores were significantly lower among medication users (M = 7.3, SD = 1.57) compared to non-users (M = 8.5, SD = 2.1), reflecting fewer delirium symptoms in the medicated group (t = -5.156, p < 0.001, 95% CI = -1.595 to -0.714). The effect size was large (Cohen's d = 0.69), suggesting a substantial difference.

**Table 4:** Comparison Among Variables (Use of Sleep Medication) CI = confidence interval; LL = lower limit; M = mean; SD = standard deviation; t = independent t-test; UL = upper limit

Variable	Yes (n = 215)	No (n = 85)	t	p	95% CI		Cohen’s d
	M ± SD	M ± SD			LL	UL	
Pittsburgh Sleep Quality Index	47.7 ± 7.8	52.1 ± 9.6	-4.109	<0.001	-6.530	-2.301	0.53
Confused Assessment Method	7.3 ± 1.57	8.5 ± 2.1	-5.156	<0.001	-1.595	-.714	0.69
Mini-Mental State Examination	19.0 ± 12.3	23.9 ± 12.4	-3.121	<0.001	-8.061	-1.827	0.40

For cognitive function, MMSE scores were significantly lower among sleep medication users (M = 19.0, SD = 12.3) compared to non-users (M = 23.9, SD = 12.4), indicating poorer cognitive performance in the medication group (t = -3.121, p < 0.001, 95% CI = -8.061 to -1.827). The effect size was small to moderate (Cohen's d = 0.40). These results suggest that while sleep medication use is associated with improved sleep quality and fewer delirium symptoms, it may be linked to reduced cognitive performance in this population.

Table 5 compares the scores on sleep quality, delirium symptoms, and cognitive performance between participants with a history of delirium or cognitive impairment (220 individuals, 73%) and those without such a history (80 individuals, 27%).

**Table 5:** Comparison Among Variables (History of Delirium or Cognitive Impairment) CI = confidence interval; LL = lower limit; M = mean; SD = standard deviation; t = independent t-test; UL = upper limit

Variable	Yes (n = 220)	No (n = 80)	t	p	95% CI		Cohen’s d
	M ± SD	M ± SD			LL	UL	
Pittsburgh Sleep Quality Index	47.1 ± 6.8	53.9 ± 10.9	-6.501	<0.001	-8.921	-4.775	0.84
Confused Assessment Method	7.2 ± 1.6	8.8 ± 1.9	-7.255	<0.001	-2.024	-1.160	0.95
Mini-Mental State Examination	17.3 ± 10.0	28.9 ± 14.7	-7.791	<0.001	-14.590	-8.705	1.02

Participants with a history of delirium or cognitive impairment reported significantly poorer sleep quality, with a lower mean PSQI score (M = 47.1, SD = 6.8) compared to those without a history (M = 53.9, SD = 10.9), yielding a large and statistically significant difference (t = -6.501, p < 0.001, 95% CI = -8.921 to -4.775; Cohen's d = 0.84).

Similarly, CAM scores were significantly lower in the history-positive group (M = 7.2, SD = 1.6) compared to the history-negative group (M = 8.8, SD = 1.9), indicating fewer delirium symptoms (t = -7.255, p < 0.001, 95% CI = -2.024 to -1.160; Cohen's d = 0.95), with a large effect size. For cognitive function, participants with a history of impairment had significantly lower MMSE scores (M = 17.3, SD = 10.0) than those without such a history (M = 28.9, SD = 14.7), indicating substantial cognitive deficits (t = -7.791, p < 0.001, 95% CI = -14.590 to -8.705; Cohen's d = 1.02), representing a huge effect size. Overall, these results highlight that a prior history of delirium or cognitive impairment is associated with significantly worse sleep quality, greater cognitive dysfunction, and fewer reported current delirium symptoms.

Table 6 presents data from the PSQI, the CAM, and the MMSE measurements, which use different sleep initiation levels. The four groups displayed a substantial statistical variation in sleep quality (F (3,296) = 9.004, p < 0.01. Participants who always had difficulty falling asleep reported the poorest sleep quality (M = 54.5, SD = 6.8), followed by those with frequent difficulty (M = 53.8, SD = 11.5). In contrast, those who never or occasionally had trouble had better sleep quality (M = 47.5 and 48.0, respectively). In terms of delirium symptoms, CAM scores also differed significantly among the groups (F (3,296) = 9.163, p < 0.01, η² = 0.085). The highest CAM scores were observed among participants reporting frequent difficulty falling asleep (M = 8.7, SD = 1.5), while the lowest scores were in the "never" group (M = 7.2, SD = 1.6), indicating increased confusion with greater sleep difficulty. For cognitive function, MMSE scores showed a highly significant difference (F (3,296) = 16.553, p < 0.01, η² = 0.144), reflecting a large effect size. Interestingly, participants with frequent difficulty falling asleep reported the highest cognitive scores (M = 30.5, SD = 16.7), while those who never had sleep difficulty had the lowest (M = 16.5, SD = 7.8). These findings indicate that increasing difficulty initiating sleep is associated with poorer sleep quality and greater confusion. However, an unexpected elevation in cognitive scores among frequent sufferers warrants further investigation, possibly due to reporting or sample variability.

**Table 6:** Comparison of Variables (Difficulty in Falling Asleep) F = F-ratio; M = mean; p = significance level; SD = standard deviation; η² = effect size

Variable	Never (n = 134); M ± SD	Occasionally (n = 108); M ± SD	Frequently (n = 41); M ± SD	Always (n = 17); M ± SD	p	F (3,296)	η²
Pittsburgh Sleep Quality Index	47.5 ± 7.5	48.0 ± 8.0	53.8 ± 11.5	54.5 ± 6.8	<0.001	9.004	0.084
Confused Assessment Method	7.2 ± 1.6	7.7 ± 1.9	8.7 ± 1.5	8.2 ± 2.1	<0.001	9.163	0.085
Mini-Mental State Examination	16.5 ± 7.8	20.6 ± 13.1	30.5 ± 16.7	25.9 ± 13.1	<0.001	16.553	0.144

Table 7 compares mean scores of the PSQI, CAM, and MMSE across different levels of self-reported feeling rested upon waking. For sleep quality, a statistically significant difference was found across groups (F (4,295) = 4.252, p < 0.01, η² = 0.055). Participants who never felt rested had the poorest sleep quality (M = 54.0, SD = 6.9), followed closely by those who rarely (M = 51.1, SD = 7.9) and sometimes (M = 50.9, SD = 9.7) felt rested. In contrast, those who always (M = 47.1, SD = 8.7) and often (M = 47.9, SD = 7.8) felt rested had better sleep quality scores.

**Table 7:** Comparison of Variables (Feeling Rested Upon Waking) F = F-ratio; M = mean; p = significance level; SD = standard deviation; η² = effect size

Variable	Never (n = 17); M ± SD	Rarely (n = 26); M ± SD	Sometimes (n = 63); M ± SD	Always (n = 77); M ± SD	Often (n = 117); M ± SD	p	F (4,295)	η^2^
Pittsburgh Sleep Quality Index	54.0 ± 6.9	51.1 ± 7.9	50.9 ± 9.7	47.1 ± 8.7	47.9 ± 7.8	0.002	4.252	0.055
Confused Assessment Method	8.8 ± 1.9	8.5 ± 2.1	7.7 ± 1.7	7.6 ± 2.0	7.2 ± 1.5	0.001	4.706	0.060
Mini-Mental State Examination	29.9 ± 20.7	23.3 ± 11.3	24.9 ± 15.0	17.5 ± 8.4	17.9 ± 10.6	<0.001	7.700	0.095

In terms of delirium symptoms, CAM scores also differed significantly among the groups (F (4,295) = 4.706, p < 0.01, η² = 0.060). The highest CAM scores were observed in participants who never felt rested (M = 8.8, SD = 1.9). In contrast, the lowest scores were seen among those who often felt rested (M = 7.2, SD = 1.5), indicating an inverse relationship between restorative sleep and confusion symptoms.

For cognitive function, MMSE scores showed a strong significant difference (F(4,295) = 7.700, p < 0.01, η² = 0.095). The highest MMSE scores were seen in the "never" group (M = 29.9, SD = 20.7), followed by "sometimes" (M = 24.9, SD = 15.0). In contrast, the lowest scores were observed in those who often or always felt rested (M = 17.9 and 17.5, respectively), suggesting a complex and possibly non-linear relationship that may reflect compensatory mechanisms, reporting bias, or differential subgroup characteristics. These findings collectively show that feeling rested upon waking is associated with better sleep quality and reduced delirium symptoms, though cognitive scores did not follow a linear pattern.

Table 8 presents survey results that measure the PSQI, CAM, and MMSE for three defined groups based on reported sleep duration each night. For sleep quality, there was a statistically significant difference among groups (F (2,297) = 15.942, p < 0.01, η² = 0.097). Participants sleeping eight to 10 hours had the poorest sleep quality scores (M = 56.4, SD = 9.7), followed by those sleeping five to seven hours (M = 52.6, SD = 8.5), while those sleeping only two to four hours reported the best sleep quality (M = 47.6, SD = 8.0), though this may reflect paradoxical reporting or sleep fragmentation. In terms of delirium symptoms, CAM scores significantly varied across groups (F (2,297) = 30.059, p < 0.01, η² = 0.168). Participants sleeping more than five hours reported higher CAM scores (M = 8.9 for both five to seven and eight- to 10-hour groups), compared to those with only two to four hours of sleep (M = 7.2, SD = 1.5), indicating that longer sleep duration was associated with greater confusion symptoms in this sample.

**Table 8:** Comparison of Variables (Duration of Sleep Per Night) F = F-ratio; M = mean; p = significance level; SD = standard deviation; η² = effect size

Variable	Two to four hours (n = 17); M ± SD	Five to seven hours (n = 26); M ± SD	Eight to 10 hours (n = 63); M ± SD	p	F (2,297)	η^2^
Pittsburgh Sleep Quality Index	47.6 ± 8.0	52.6 ± 8.5	56.4 ± 9.7	<0.001	15.942	0.097
Confused Assessment Method	7.2 ± 1.5	8.9 ± 2.3	8.9 ± 1.5	<0.001	30.059	0.168
Mini-Mental State Examination	18.1 ± 10.9	26.9 ± 16.0	31.1 ± 8.9	<0.001	19.652	0.117

For cognitive function, MMSE scores also showed a statistically significant difference (F (2,297) = 19.652, p < 0.01, η² = 0.117). The highest cognitive scores were observed among those sleeping eight to 10 hours (M = 31.1, SD = 8.9), followed by five to seven hours (M = 26.9, SD = 16.0), and the lowest scores in those sleeping two to four hours (M = 18.1, SD = 10.9), suggesting a positive association between longer sleep duration and better cognitive performance. Overall, the results suggest a complex relationship where longer sleep duration was linked to improved cognitive function but also with greater confusion symptoms and poorer perceived sleep quality, potentially reflecting underlying health or neurological factors.

Table 9 demonstrates the results of a multiple regression analysis predicting CAM scores using PSQI scores and MMSE scores as predictors.

**Table 9:** Multiple Regression Analysis Predicting Confusion Assessment Method (CAM) Scores Using Mini-Mental State Examination (MMSE) and Pittsburgh Sleep Quality Index (PSQI) B = coefficient; β = standardized coefficient; CI = confidence interval; LL = lower limit; SE = standard error; UL = upper limit p < 0.01 is considered significant.

Variable	B	95% Cl		SE	β	p
		LL	UL			
Constant	5.063	3.959	6.168	0.561	-	<0.001
Pittsburgh Sleep Quality Index	0.027	0.001	0.053	0.013	0.127	0.04
Mini-Mental State Examination	0.061	0.043	0.079	0.009	0.419	<0.001

Both predictors were found to be statistically significant. As indicated by higher PSQI scores, Poorer sleep quality was a significant but modest predictor of increased confusion symptoms (B = 0.027, β = 0.127, p = 0.04), with the 95% confidence interval ranging from 0.001 to 0.053.

Cognitive functioning, measured by MMSE, emerged as a stronger and highly significant predictor (B = 0.061, β = 0.419, p < 0.001), with a 95% confidence interval of 0.043 to 0.079, indicating that lower cognitive performance was associated with greater confusion symptoms. The model suggests that poorer sleep quality and impaired cognition contribute significantly to increased delirium-related symptoms in the sample.

**Figure 1:**
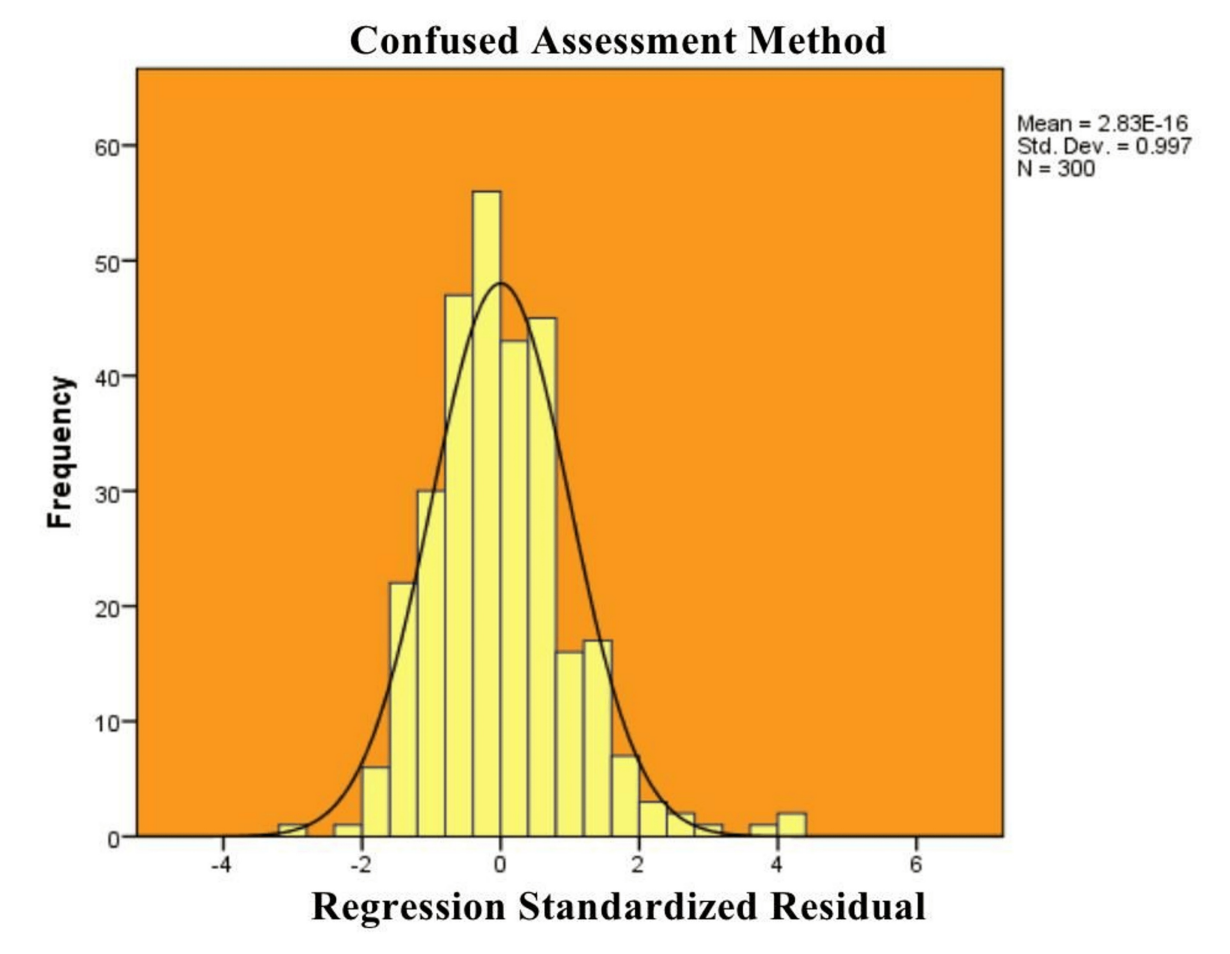
Histogram of Standardized Residuals From the Regression Analysis of the Confused Assessment Method

Figure 1 presents a histogram of regression standardized residuals for the "Confused Assessment Method," illustrating a near-normal distribution pattern. The mean is close to zero, and the standard deviation is approximately 1, suggesting that the residuals are symmetrically distributed and the regression model is well-fitted. The visual representation supports the assumption of normality in the residuals, which is a key requirement for linear regression analysis.

**Figure 2:**
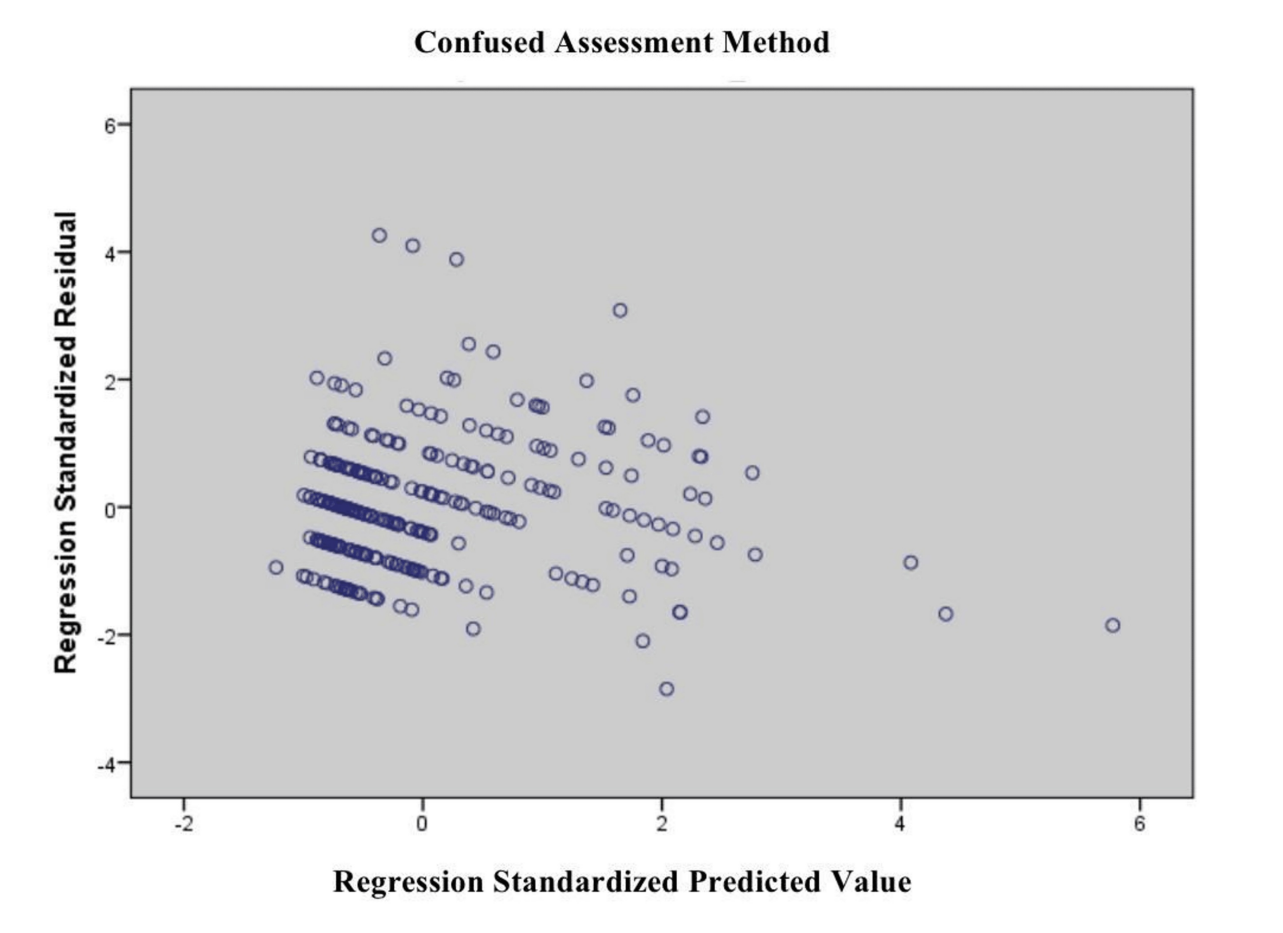
Scatterplot of Standardized Residuals vs. Predicted Value for Regression of Confused Assessment Method

Figure 2 presents a residual scatter plot for the "Confused Assessment Method," revealing signs of heteroscedasticity. The residuals exhibit a fan-shaped spread, becoming wider with increasing predicted values. This pattern indicates unequal variance in residuals, suggesting that the model may violate the homoscedasticity assumption, implying potential issues with prediction consistency and model reliability across the range of predicted scores.

Table 10 illustrates the associations between difficulty in sleeping and two sleep-related variables: feeling rested upon waking and duration of sleep per night, using the chi-square test for categorical comparisons.

**Table 10:** Descriptive Statistics of Demographic Variables (Difficulty in Sleeping, Feeling Rested Upon Waking, and Duration of Sleep per Night) f = frequency; p = level of significance p-Values are calculated using the chi-square test. The significance level is set at p < 0.05.

Variables	f	Feeling rested upon waking					p	χ²	Duration of sleep per night			p	χ²
		Never	Rarely	Sometimes	Always	Often			Two to four hours	Five to seven hours	Eight to 10 hours		
Difficulty in sleeping	-	-	-	-	-	-	0.02	24.2	-	-	-	0.001	22.9
Never	134	1	7	33	41	52	-	-	109	21	4	-	-
Occasionally	108	11	15	15	23	44	-	-	84	20	4	-	-
Frequently	41	3	3	12	8	15	-	-	24	8	9	-	-
Always	17	2	1	3	5	6	-	-	13	3	1	-	-

A statistically significant association was observed between difficulty in sleeping and feeling rested upon waking (χ² = 24.2, p = 0.02). Among participants who never had trouble sleeping (n = 134), the majority reported feeling rested either often (n = 52) or always (n = 41). In contrast, those who frequently or consistently had difficulty sleeping were likelier to report never or rarely feeling rested upon waking, indicating a strong link between sleep initiation issues and reduced restorative sleep. An even stronger and highly significant association was found between sleep difficulty and sleep duration per night (χ² = 22.9, p = 0.001). Most participants with no sleep difficulty (n = 134) slept five to seven hours per night (n = 109), while those with frequent or constant difficulty were more likely to report shorter sleep durations (two to four hours), with very few reaching eight to 10 hours. This highlights that individuals with more frequent sleep difficulties tend to experience shorter sleep durations and less recovery upon waking.

These results underscore the interconnectedness of subjective sleep quality, perceived restfulness, and sleep duration in older adults.

Table 11 explores the relationship between pre-existing medical conditions and two key sleep-related variables: feeling rested upon waking and sleep duration per night, using chi-square tests for categorical comparison.

**Table 11:** Descriptive Statistics of Demographic Variables (Pre-existing Medical Conditions, Feeling Rested Upon Waking, and Duration of Sleep per Night) f = frequency; p = level of significance p-Values are calculated using the chi-square test. The significance level is set at p < 0.05.

Variables	f	Feeling rested upon waking					p	χ²	Duration of sleep per night			p	χ²
		Never	Rarely	Sometimes	Always	Often			Two to four hours	Five to seven hours	Eight to 10 hours		
Pre-existing medical conditions	-	-	-	-	-	-	0.02	35.8	-	-	-	<0.001	52.3
Hypertension	99	4	8	20	32	35	-	-	83	13	3	-	-
Diabetes	93	2	7	17	27	40	-	-	79	12	2	-	-
Cardiac disease	44	3	1	6	8	26	-	-	35	7	2	-	-
Respiratory diseases	34	5	5	10	4	10	-	-	23	7	4	-	-
Cognitive impairment/dementia	18	2	3	7	4	2	-	-	4	8	6	-	-
Depression/anxiety	12	1	2	3	2	4	-	-	6	5	1	-	-

A statistically significant association was found between pre-existing medical conditions and feeling rested upon waking (χ² = 35.8, p = 0.02). Participants with hypertension (n = 99) and diabetes (n = 93) most frequently reported feeling rested either often or always (n = 35 and 40, respectively). In contrast, those with respiratory diseases (n = 34) and cognitive impairment/dementia (n = 18) were more likely to report never or rarely feeling rested. This suggests that certain chronic conditions, particularly those affecting respiratory and cognitive function, may impair subjective sleep recovery.

A highly significant association was also observed between pre-existing medical conditions and sleep duration per night (χ² = 52.3, p < 0.001). Participants with hypertension and diabetes predominantly reported sleeping five to seven hours per night (n = 83 and 79, respectively). Conversely, individuals with cognitive impairment/dementia showed more varied sleep patterns, with notable proportions reporting both short (two to four hours) and long (eight to 10 hours) sleep durations. Participants with respiratory disease (n = 34) and depression/anxiety (n = 12) had more irregular sleep patterns, including a higher frequency of shorter sleep durations.

These findings indicate that chronic health conditions are closely linked to variations in perceived sleep quality and sleep duration, with certain conditions, such as cognitive impairment and respiratory disease, showing the most disrupted sleep profiles.

## Discussion

The results of our study establish that sleep quality and cognitive functioning are significant predictors of delirium, where poor sleep quality and lower cognitive performance cause greater confusion symptoms. The research results indicate a two-way relationship where previous delirium episodes lead to impaired sleep quality and cognitive problems, although participants show limited delirium symptoms at present. The poorest sleep quality was reported by those who had difficulty falling asleep, never felt rested, and slept eight to 10 hours a night. An increase in confusion was observed in those who had difficulty falling asleep, often felt rested, and slept longer. Participants with longer sleep duration and difficulty initiating sleep reported higher cognitive scores. The lowest cognitive scores were observed in those who often felt rested. Those who did not have difficulty sleeping slept for longer durations and felt well-rested upon waking up. Participants with certain chronic conditions affecting respiratory and cognitive functioning rarely felt rested. Those with depression/anxiety and respiratory symptoms had shorter sleep duration.

The analysis by He et al., combined with the findings of Todd et al., discovered that disrupted sleep patterns lead to POD development [19,20]. This outcome, in line with our study, may be due to various reasons, including neuroinflammation, dysfunction of the blood-brain barrier, and buildup of amyloid beta proteins [21]. Literature has previously indicated the involvement of acetylcholine and serotonin dysregulation in delirium pathophysiology, with the implication that delirium can be caused by numerous overlapping neurotransmitter dysfunctions [22].

A previous history of delirium was associated with worse sleep quality and poor cognitive performance in our study. Delirium may be independently associated with cognitive decline in surgical and nonsurgical groups, as suggested by Goldberg et al. [23]. EEG in delirium patients has shown slow brain activity, as shown by increased delta and theta waves. This manifests as impaired cortical function and is associated with the severity of cognitive disturbances [24]. Another study on ICU survivors found that those who experienced delirium during their stay at the hospital reported poorer sleep quality post-discharge. A finding that highlights the potential long-term relationship between delirium and sleep disruption, which may also be relevant to adults undergoing elective surgery [25].

Delirium symptoms significantly increased in those who had difficulty falling asleep, often felt rested, and slept longer. A study by Nameth et al. revealed a U-shaped relation between the development of delirium and duration of sleep, where those who slept >8 hours showed a greater risk of developing delirium [26]. In another study, sleep quality did not predict the occurrence of delirium in 24 hours, suggesting that patients' perceptions of feeling rested may not accurately reflect their risk for delirium [27].

The results of our study also highlight how chronic medical conditions affect sleep patterns and duration. Literature shows that aging and chronic health conditions, such as heart failure and respiratory conditions, are associated with decreased total sleep time and reduced sleep efficiency [28-30]. A retrospective study at Aga Khan University Hospital documented a 22% prevalence of delirium in elderly inpatients, and identified infection, poor functionality, and the use of psychoactive drugs as significant risk factors. The report emphasizes the necessity of enhanced screening and management of delirium in Pakistani healthcare facilities [31]. A study shows this is due to many factors, including the symptoms associated with the illness. Patients with respiratory illnesses who present with coughing and wheezing have higher rates of insomnia and daytime sleepiness. Sleep fragmentation was also observed in such patients, resulting in poor sleep quality. The participants demonstrated signs of anxiety and depression, which primarily appeared through breathing challenges associated with their respiratory health [32].

Limitations

This study, although intended as a prospective cohort investigation, has several limitations. While the prospective design enables temporal ordering between preoperative sleep disturbances and POD, causality cannot be conclusively inferred without extended follow-up and control in a controlled experiment. Secondly, the assessment was based on self-report tools like the PSQI, CAM, and MMSE, which have possibly introduced recall or reporting bias and influenced the precision of the assessment of delirium and sleep disturbances. Even though trained personnel administered the CAM, a potential diagnostic bias exists. Another limitation is that Participants who had trouble initiating sleep unexpectedly had higher MMSE scores, which may indicate reporting bias or unmeasured confounders like education or occupation; hence, adjusted analyses in future studies are essential. The exclusion of patients with pre-existing cognitive impairment or dementia, necessary as it was to study clarity, could have skewed the outcomes if cognitive impairment is a major variable affecting the risk of delirium; this limitation should be acknowledged when interpreting findings. The research applied a non-probability sampling technique, which could have resulted in selection bias and lower generalizability of results. Secondly, though the ultimate sample size of 300 respondents was sufficient for preliminary analysis, it was less than the desired 371 respondents and could have affected statistical power and the stability of conclusions. Finally, no control was exercised for potential confounding factors like comorbidities or type of surgery, which may have affected the observed associations.

Future directions

In the future, the samples used should be larger and more representative to strengthen the study’s findings. Although this research was based on a prospective cohort design, robust findings could be obtained by evaluating patients over longer postoperative periods. If researchers control for type and duration of surgery, types of anesthesia given, and drug use, it will become clearer how these factors help in the development of delirium. Seeing improvements in sleep before surgery might provide a solution for reducing later delirium. Lastly, standardized training for healthcare professionals to assess and manage delirium will enhance early detection and treatment results.

## Conclusions

Research reveals a direct association between preoperative sleep disturbances and the subsequent development of delirium in patients undergoing elective surgical operations. As the surgical population increasingly includes middle-aged and older adults, optimizing modifiable risk factors, such as sleep quality, becomes essential for better outcomes. Detection and treatment of sleep disturbances before surgery could provide a cost-saving, non-invasive means of minimizing the risk of POD. These results highlight the imperative for standard routine screening and prompt intervention in preoperative patients with sleep problems. These results highlight the importance of standardized routine screening and timely interventions on sleep issues for preoperative patients. Further studies are necessary to confirm these findings in support of integrating sleep-centered strategies into perioperative care practice guidelines, leading to better surgical outcomes for adults.
